# Validation of a Decision Rule for Selective TSH Screening in Atrial Fibrillation

**DOI:** 10.5811/westjem.2014.11.23490

**Published:** 2015-01-05

**Authors:** Shawna D. Bellew, Rajat Moman, Christine M. Lohse, Erik P. Hess, M. Fernanda Bellolio

**Affiliations:** * Mayo Clinic, Department of Emergency Medicine, Rochester, Minnesota; † Mayo Medical School, Rochester, Minnesota; ‡ Mayo Clinic, Department of Health Sciences Research, Division of Biostatistics, Rochester, Minnesota; § Mayo Clinic, Robert D. and Patricia E. Kern Center for the Science of Health Care Delivery, Rochester, Minnesota

## Abstract

**Introduction:**

Atrial fibrillation (AF) is the most common cardiac dysrhythmia. Current guidelines recommend obtaining thyroid-stimulating hormone (TSH) levels in all patients presenting with AF. Our aim was to investigate the utility of TSH levels for emergency department (ED) patients with a final diagnosis of AF while externally validating and potentially refining a clinical decision rule that recommends obtaining TSH levels only in patients with previous stroke, hypertension, or thyroid disease.

**Methods:**

We conducted a retrospective, cross-sectional study of consecutive patients who presented to an ED from January 2011 to March 2014 with a final ED diagnosis of AF. Charts were reviewed for historical features and TSH level. We assessed the sensitivity and specificity of the previously derived clinical decision rule.

**Results:**

Of the 1,964 patients who were eligible, 1,458 (74%) had a TSH level available for analysis. The overall prevalence of a low TSH (<0.3μIU/mL) was 2% (n=36). Elevated TSH levels (>5μIU/mL) were identified in 11% (n=159). The clinical decision rule had a sensitivity of 88.9% (95% CI [73.0–96.4]) and a specificity of 27.5% (95% CI [25.2–29.9]) for identifying a low TSH. When analyzed for its ability to identify any abnormal TSH values (high or low TSH), the sensitivity and specificity were 74.4% (95% CI [67.5–80.2]) and 27.3% (95% CI [24.9–29.9]), respectively.

**Conclusion:**

Low TSH in patients presenting to the ED with a final diagnosis of AF is rare (2%). The sensitivity of a clinical decision rule including a history of thyroid disease, hypertension, or stroke for identifying low TSH levels in patients presenting to the ED with a final diagnosis of atrial fibrillation was lower than originally reported (88.9% vs. 93%). When elevated TSH levels were included as an outcome, the sensitivity was reduced to 74.4%. We recommend that emergency medicine providers not routinely order TSH levels for all patients with a primary diagnosis of AF. Instead, these investigations can be limited to patients with new onset AF or those with a history of thyroid disease with no known TSH level within three months.

## INTRODUCTION

Atrial fibrillation (AF) is the most common cardiac dysrhythmia, affecting an estimated three million persons in the United States, a number that is expected to increase to 7.5 million by the year 2050.^1^,^2^ Associated with heart failure and stroke, AF represents a significant contributor to mortality, morbidity, and healthcare expenditures.^3^,^4^ AF accounts for 0.5% of all emergency department (ED) visits, a setting in which initial diagnosis and management often occurs.^5^,^6^

Initial management of AF includes ruling out reversible causes and contributors to the condition, including thyroid dysfunction.^7^–^9^ Atrial fibrillation has long been observed to be a sequela of hyperthyroidism. This relationship has been described in both clinical and subclinical hyperthyroidism, with overt hyperthyroidism conferring up to a five-fold increase in the relative risk of developing AF.^10^–^12^ Patients with subclinical hyperthyroidism or even high normal thyroid function have been shown to be significantly more likely to develop AF, with as much as a three-fold increase in risk.^13^,^14^ More controversial is the relationship of hypothyroidism and AF. While early studies suggested an association between the two conditions,^15^–^17^ others have indicated that hypothyroidism might actually be protective against developing AF.^18^

Although many studies have reported an increased incidence of AF in patients with thyroid disease, particularly hyperthyroidism, far fewer studies have focused on the incidence of thyroid disease in those with AF. Published studies indicate that while abnormal thyroid stimulating hormone (TSH) levels are present in as many as 16.6% of patients with atrial fibrillation,^19^ the incidence of clinically significant thyroid disease is likely closer to 2%.^20^ Therefore, the utility of routine testing has been called into question.

In 2010, Bruccelletti et al. published the results of a cross-sectional observational study of 433 patients admitted to an ED observation unit for new-onset atrial fibrillation who underwent thyroid function tests.^21^ Recursive partitioning was performed in an effort to identify clinical characteristics associated with a TSH <0.35μIU/mL. From this analysis, a model was proposed that recommended obtaining TSH levels only in patients with any one of the following: previous cerebrovascular disease, hypertension, or thyroid disease. When applied to the derivation patient population, this model had a sensitivity of 93% and a specificity of 31%. Application of the model could potentially have avoided 30% of TSH evaluations in the study population.

Given the expanding prevalence of AF, limiting the acquisition of TSH levels could be a small but substantial step towards decreasing the cost of care for this patient population. With this end objective in mind, we aimed to externally validate this clinical decision rule with a secondary goal of identifying any further predictors of an abnormal TSH in our population for further refinement of the model. Finally, we set out to describe the incidence of TSH abnormalities in a large sample of ED patients with a diagnosis of AF, therefore exploring the yield of this commonly ordered laboratory investigation.

## METHODS

### Study Design and Setting

We performed a retrospective, cross-sectional study of all the patients who presented to an academic tertiary care emergency department (ED) with 73,000 annual patient visits with an ED diagnosis of atrial fibrillation. Consecutive patients presenting from January 3, 2011 to March 16, 2014 with a final diagnosis of atrial fibrillation were included. Patients were excluded if they did not consent to having their medical records reviewed for research purposes. The institutional review board approved the research protocol.

### Data Processing

Electronic medical records (EMR) with a final diagnosis of atrial fibrillation after the ED visit were extracted by a data quality analyst. For each patient, the following data were extracted from the EMR: date, time of visit, patient age, gender, diagnosis, disposition, vital signs, and medications administered. Then, a focused chart review was performed by a resident physician and a medical student. The following data were extracted into a Microsoft Excel (Microsoft Corporation, Redmond, WA) spreadsheet: history of hypertension, heart failure, diabetes mellitus, cerebrovascular accident or transient ischemic attack, thyroid disease, and TSH level (μIU/mL) within 24 hours of ED presentation or within 30 days of ED presentation. For each patient, the presence or absence of historical features was abstracted from the additive problem list feature of the EMR, which often contains prior primary care visits as well as the identified ED visit. Reviewers agreed upon a predetermined definition of the historical features abstracted, in which heart failure was considered present regardless of preserved systolic function if a diagnosis was made by a cardiologist, hypertension was considered present if the patient carried a previous diagnosis of hypertension, cerebrovascular disease included a diagnosis of stroke, cerebrovascular accident, or transient ischemic attack, and thyroid disease included hypothyroidism or hyperthyroidism. We used these historical features to calculate the Congestive heart failure, Hypertension, Age ≥75, Diabetes mellitus, Prior Stroke or Transient ischemic attack or Thrombeombolism (CHADS2) score for each patient. The CHADS2 score is a clinical prediction rule that generates an estimated yearly risk of stroke in patients with AF. Patients with higher scores are more likely to experience stroke. Statistical analyses were performed by a biostatistician using the SAS software package (SAS Institute, Cary, NC).

### Data Analysis

The main outcome measures were the test characteristics of the clinical decision rule including sensitivity, specificity, positive predictive value (PPV), negative predictive value (NPV), positive likelihood ratio (+LR) and negative likelihood ratio (-LR) for identifying an abnormal TSH level. Analysis of these characteristics was performed separately to assess the performance of the rule to identify a low TSH level or any abnormal TSH level. In addition to determining the performance characteristic of the decision rule, we further analyzed each feature separately for its independent correlation to low or abnormal TSH levels.

We summarized continuous data were summarized with means, medians, and IQRs, while categorical data were summarized with frequency counts and percentages. Comparisons of characteristics of patients with and without a TSH available for analysis were evaluated using Wilcoxon rank sum and chi-square tests. Only patients who had a TSH available on chart abstraction were included in the estimation of the sensitivity, specificity, +LR, -LR, PPV, and NPV of the clinical decision rule. Comparisons of features by TSH level (≥0.3μIU/mL versus <0.3μIU/mL and normal versus abnormal) were evaluated using Wilcoxon rank sum, chi-square, and Fisher exact tests. We defined an abnormal TSH level as being either <0.3 or >5μIU/mL. These cutoffs were based on the institution’s laboratory definitions of normal. All tests were two-sided and we considered p-values <0.05 statistically significant.

## RESULTS

From January 2011 to March 2014, 2,071 patients presented to the ED with a final diagnosis of atrial fibrillation. One hundred and seven patients declined consent for charts review. Therefore, 1,964 patients were included. The features of these patients are summarized in Table 1.

A TSH level was available for 1,458 (74%) of these patients, who were therefore included in the analysis of the diagnostic performance of the clinical decision rule. Seventy-one percent (n=1032) of these values were obtained within 24 hours of ED presentation and the remaining within 30 days of presentation. Patients with a TSH level available were more likely to be female (47% vs. 41%; p=0.036), more likely to have a history of thyroid disease (20% vs. 9%; p <0.001), and had higher heart rates (median 120 vs. 115; p <0.001) compared to patients without a TSH level measured. Seventy-three percent (n=1063) of the patients with a TSH level available for analysis met at least one of the criteria for the clinical decision rule (presence of cerebrovascular disease, hypertension, or thyroid disease). Seventy percent (n=353) of the 506 patients without a TSH level available met the criteria (p=0.17).

Of the 1,458 patients with a TSH available for analysis, 36 (2%) had a low TSH level (<0.3μIU/mL), 1263 (87%) a normal TSH level (between 0.3 and 5.0μIU/mL), and 159 (11%) a high TSH level (>5.0μIU/mL). Table 2 shows the association between clinical features and low TSH levels. There was no significant association between temperature, heart rate, blood pressure, or CHADS2 score and a low TSH level. Female sex and a history of thyroid disease were significantly associated with a low TSH level. When these features were analyzed for their association with any TSH abnormality, the findings were largely the same (illustrated in Table 3).

Applying the rule, 1063 (72.9%) of the 1,458 patients met criteria for having a TSH level drawn, which identified 32 (88.9%) of the 36 patients with an abnormally low TSH level. This resulted in the following performance characteristics: sensitivity 88.9% (95% CI [73.0–96.4]), specificity 27.5% (95% CI [25.2–29.9]), positive LR 1.23 (95% CI [1.09–1.38]), negative LR 0.40 (95% CI [0.16–1.02]), PPV 3.0% (95% CI [2.1–4.3]), and NPV 99.0% (95% CI [97.2–99.7]) (Figure, Table 4). When both abnormally low and high TSH levels were analyzed as the outcome, the criteria identified 145 of the 195 patients with abnormal levels. The clinical decision rule had the following diagnostic performance for detecting any TSH abnormality: sensitivity 74.4% (95% CI [67.5–80.2]), specificity 27.3% (95% CI [24.9–29.9]), positive LR 1.02 (95% CI [0.94–1.12]), negative LR 0.94 (95% CI [0.74–1.20]), PPV 13.6% (95% CI [11.7–15.9]), and NPV 87.3% (95% CI [83.6–90.4]) (Figure, Table 4).

## DISCUSSION

Atrial fibrillation (AF) affects up to 8% of the U.S. population by the age of 80 and costs an estimated 26 billion dollars per year.^22^,^23^ AF is associated with hypertension, diabetes mellitus, heart failure, obstructive sleep apnea, and obesity. Most commonly, AF is thought to result from histopathologic changes of the atrial walls, resulting in aberrant conduction. In animal studies, both hyperthyroidism and hypothyroidism result in interstitial fibrosis of the atrial walls and therefore AF.^24^,^25^

Hypothyroidism occurs in approximately 4% of the population, becoming increasingly common with advanced age.^26^ Hyperthyroidism is less common, affecting less than 0.5%. Thus, despite the increased risk of developing AF conferred by hyperthyroidism, the incidence of hyperthyroidism in AF remains small, reported from 0.7% to 5.2%.^27^–^29^ Meanwhile, hypothyroidism is found in 8–15% patients with AF.^16^,^17^,^28^,^29^ There is some controversy as to whether hypothyroidism plays a contributory role in the development of AF versus simply tending to occur in the same patient population, namely the elderly.^30^ A recent cohort study of Framingham heart study participants found no significant association with hypothyroidism and increased risk of AF over 10-years.^31^ However, there is a plausible mechanism for hypothyroidism contributing to AF, given that it does seem to contribute to associated conditions, namely hypertension, atherosclerosis, and heart failure.^7^

In our study, 74% of patients had a TSH level available for analysis (52% drawn within 24 hours of ED presentation and another 22% with levels drawn within 30 days of presentation). These patients were more likely to be female (47% vs. 41%; p=0.036) and were more likely to have a history of thyroid disease (20% vs. 9%; p<0.001) than patients who had no available level. It is not surprising that many patients did not have a TSH ordered, given that our institution possesses a highly integrated EMR that captures much of the surrounding primary care, and therefore many patients likely already had an available recent TSH level.

The incidence of abnormal TSH levels in our study was 13%. Two percent of patients had low TSH levels (<0.3μIU/mL) and 11% had high TSH levels (>5μIU/mL). Therefore, a provider would have to screen 50 patients presenting with AF to identify one patient with hyperthyroidism. These incidences are similar to those previously reported, though the prevalence of hyperthyroidism in our study population was markedly less than the 10.8% reported by Bruccelleti et al.^21^ Prior to this study, there were few published studies reporting the prevalence of these findings specific to the ED setting or patient population.^19^

We found a high incidence of patients with a history of thyroid disease (18%). This was likely due to our broad definition of thyroid disease, as we included any past diagnosis available in the EMR of thyroid abnormality including subclinical hyperthyroidism or hypothyroidism. However, our measured incidences of TSH abnormalities were consistent with that previously reported. Therefore, our study population is likely representative of the general population.

When externally validating the previously derived rule proposed by Bruccelleti et al in 2010, we found a mildly lower sensitivity and specificity for hyperthyroidism (89.9%; 27.5%) when compared that originally reported (93%; 31%). Given the low incidence of hyperthyroidism, the negative predictive value remained high (99%). Though the existence of any causal relationship between hypothyroidism and AF remains questionable, detecting underlying hypothyroidism could have implications for downstream care. Therefore, we also analyzed the ability of the rule to detect any TSH abnormality. Under these constraints, the rule was predictably less sensitive (74.4%), with a lower negative predictive value (87.3%) than for predicting hyperthyroidism alone.

Notably, we did not limit our study population to new onset atrial fibrillation, as was done by Bruccelleti et al. Based on provider final diagnosis, approximately 13% of our sample was new onset atrial fibrillation. As supported by our data, TSH levels are ordered in patients with a primary ED diagnosis of AF regardless of whether the impetus for the visit is chronic AF with a rapid ventricular rate, paroxysmal symptomatic AF, or newly discovered AF. Therefore, for the rule to have maximal economic impact, it would have to be sufficiently sensitive in all comers with a primary diagnosis of AF. Perhaps, patients with new onset AF are more likely to have a secondary cause, thus increasing the positive predictive value of the decision rule for that specific subset of patients. However, the sensitivity of the rule, which is the overriding concern of a screening tool, should not be altered.

In order to refine the clinical decision rule, we extracted additional data from the EMR, including vital signs, CHADS2 features (a history of heart failure, age, diabetes mellitus, hypertension and stroke or TIA). We then looked for a correlation between any of these features and TSH abnormalities. Though we originally hypothesized that younger patients, perhaps with a particular pattern of vital sign abnormalities, would be more likely to have underlying thyroid dysfunction as a contributing factor to AF, we did not identify any further features suggestive of possible refinements for previously derived rule.

## LIMITATIONS

There are several limitations to this validation study. Foremost among these is the retrospective nature of our investigation, which made it impossible to ensure universal TSH sampling. For this reason, we have no way of knowing what factors led providers to order or forgo ordering TSH levels. Further, the retrospective nature of the study necessitated use of the cumulative EMR past medical history, which may have been incomplete. However, given the highly integrated nature of our healthcare system, it is also likely that we had more information than would typically be available by interview alone. We did not isolate our analysis to new-onset or recent onset AF. Instead, we chose to include all patients presenting with AF in order to maximize clinical applicability. This is a single center study, which is potentially problematic as the incidence of thyroid disease varies geographically. Lastly, we were unable to capture the clinical significance of the identified TSH abnormalities, since we do not know whether these led to alterations in management.

Previous studies investigating the value of TSH screening in AF have used various cutoffs to help delineate the difference between technical and clinically important TSH abnormalities. Typically, the threshold for defining clinically significant hyperthyroidism has ranged from 0.1μIU/mL to as high as 0.3μIU/mL in later studies following the publication of evidence of a relationship between subclinical hyperthyroidism in AF.

For hypothyroidism, a threshold of as high as 20μIU/mL has been used.^29^ When these more specific, less sensitive cutoffs were used on our data, the clinical performance metrics of the rule were largely unchanged. The resulting sensitivity, specificity, positive predictive value, and negative predictive value for TSH <0.1 or >20μIU/mL was calculated to be 88.9% (95% CI [69.7–97.1]), 27.4% (25.1–29.8), 2.3% (1.5–3.4), and 99.2% (97.6–99.8), respectively. The lack of universally accepted cutoffs for defining clinically significant TSH abnormalities complicates the interpretation of the literature in this field and the management of TSH abnormalities in clinical practice.

The utility of obtaining routine TSH levels and moreover applying the Bruccelleti rule is dependent on the degree of importance clinicians place on identifying abnormalities in thyroid function. Presumably, the primary objective in identifying abnormal thyroid function is to diagnose underlying hyperthyroidism. Hyperthyroidism has a specific management implication in AF. About two-thirds of patients will spontaneously convert to sinus rhythm upon thyroid normalization, and current guidelines suggest that a euthyroid state should be reached prior to cardioversion.^7^,^32^,^33^ There are no specific guidelines addressing management of AF in the setting of hypothyroidism.

## CONCLUSION

While TSH abnormalities in emergency department patients with a primary diagnosis of AF are common (13%), low TSH is rare (2%). Only hyperthyroidism has a direct management implication in the acute management of AF. We externally validated a clinical decision rule which included criteria of history of thyroid disease, hypertension, or cerebrovascular disease for its ability to predict TSH abnormalities in patients presenting to the emergency department with atrial fibrillation. We found a slightly lower sensitivity and specificity for hyperthyroidism (89.9%; 27.5%) compared to that originally reported by Bruccelleti et al. (93%; 31%).^21^ We recommend that emergency medicine providers not routinely order TSH levels for all patients presenting with a primary diagnosis of AF. Instead, these investigations can be limited to patients with new onset AF or those with a history of thyroid disease with no known TSH level within three months.

## Figures and Tables

**Figure:**
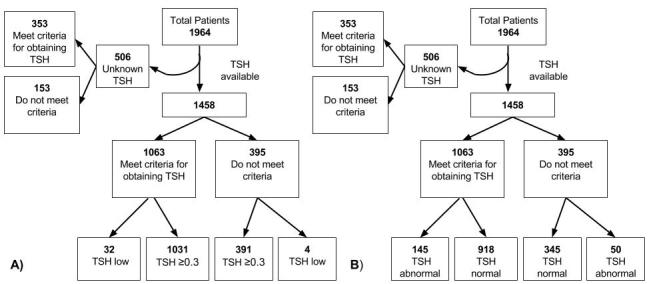
Flow diagram illustrating validation of a clinical decision rule including a history of thyroid disease, cerebrovascular disease, or hypertension for identifying (A) low TSH (<3μIU) or (B) any TSH abnormality (<0.3 or >5μIU/mL). *TSH*, thyroid-stimulating hormone

**Table 1:** Features of all eligible patients, N=1,964, in a study of the utility of TSH levels for emergency department patients with a final diagnosis of atrial fibrillation.

Feature	Mean (Median; IQR)
Age in years	68.9 (70;61–79)
Gender	N (%)
Female	886 (45)
Male	1078 (55)
Congestive heart failure	494 (25)
Diabetes mellitus	375 (19)
Cerebrovascular disease	198 (10)
Hypertension	1291 (66)
Thyroid disease	345 (18)
CHADS2 score	
0	385 (20)
1	585 (30)
2	536 (27)
3	277 (14)
4	125 (6)
5	46 (2)
6	10 (1)
TSH μIU/mL (N=1458)	
<0.1	24 (2)
<0.3	36 (2)
0.3–5.0	1263 (87)
>5.0	159 (11)
>20.0	3 (<1)

*CHADS2*, Congestive heart failure, Hypertension, Age ≥75, Diabetes mellitus, Prior Stroke or Transient ischemic attack or Thrombeombolism; *TSH*, thyroid-stimulating hormone

**Table 2:** The predictive value of features for a low TSH level (≥0.3 versus <0.3μIU), N=1,458.

	TSH level [N (%)]			LR (95% CI)	
		
Feature	≥0.3μIU (n=1,422)	<0.3μIU (n=36)	p-value	+LR*	−LR*
Age†					
<75	909 (64)	19 (53)	0.17	0.83 (0.60–1.13)	1.31 (0.92–1.85)
≥ 75	513 (36)	17 (47)	0.17	0.83 (0.60–1.13)	1.31 (0.92–1.85)
Sex					
Female	654 (46)	24 (67)	0.14	1.45 (1.13–1.84)	1.45 (1.13–1.84)
Male	768 (54)	12 (33)	0.14	1.45 (1.13–1.84)	1.45 (1.13–1.84)
Congestive heart failure	363 (26)	8 (22)	0.65	0.87 (0.47–1.61)	1.04 (0.88–1.24)
Diabetes mellitus	274 (19)	6 (17)	0.70	0.85 (0.41–1.81)	1.03 (0.89–1.19)
Cerebrovascular disease	134 (9)	2 (6)	0.57	0.59 (0.15–2.29)	1.04 (0.96–1.13)
Hypertension	932 (66)	21 (58)	0.37	0.89 (0.67–1.18)	1.21 (0.82–1.78)
Thyroid disease	275 (19)	23 (64)	<0.001	3.30 (2.53–4.32)	0.45 (0.29–0.69)
CHADS2 score					
0	277 (19)	10 (28)	0.55		
1	435 (31)	8 (22)	0.55		
2	380 (27)	11 (31)	0.55		
3	208 (15)	4 (11)	0.55		
4	85 (6)	2 (6)	0.55		
5	31 (2)	0	0.55		
6	6 (<1)	1 (3)	0.55		

* Positive and negative likelihood ratios for predicting a low TSH level (<3μIU).

† Mean age for ≥0.3μIU was 68.7; median 70, IQR 60–79. Mean age for <0.3μIU was 68.8; median 69; IQR 60–81. p-value 0.96.

*TSH*, thyroid-stimulating hormone; +*LR*, positive likelihood ratio; −*LR*, negative likelihood ratio; *CHADS2*, Congestive heart failure, Hypertension, Age ≥75, Diabetes mellitus, Prior Stroke or Transient ischemic attack or Thrombeombolism

**Table 3:** The predictive value of features for an abnormal TSH level (<0.3 or >5μIU/mL), N=1,458.

	TSH level [N (%)]			LR (95% CI)	
		
Feature	Normal (n=1,263)	Abnormal (n=195)	p-value	+LR*	−LR*
Age					
<75	797	131	0.27	1.06 (0.96–1.18)	0.89 (0.73–1.09)
≥ 75	466	64	0.27	1.06 (0.96–1.18)	0.89 (0.73–1.09)
Sex					
Female	572 (45)	106 (54)	0.18	1.20 (1.04–1.38)	0.83 (0.71–0.97)
Male	691 (55)	89 (46)	0.18	1.20 (1.04–1.38)	0.83 (0.71–0.97)
Congestive heart failure	324 (26)	47 (24)	0.64	0.94 (0.72–1.23)	1.02 (0.94–1.11)
Diabetes mellitus	249 (20)	31 (16)	0.21	0.81 (0.57–1.13)	1.04 (0.99–1.11)
Cerebrovascular disease	123 (10)	13 (7)	0.17	0.68 (0.39–1.19)	1.03 (1.00–1.07)
Hypertension	846 (67)	107 (55)	<0.001	0.82 (0.72–0.94)	1.37 (1.17–1.60)
Thyroid disease	221 (18)	77 (39)	<0.001	2.25 (1.83–2.79)	0.73 (0.65–0.82)
CHADS2 score					
0	231 (18)	56 (29)	0.002		
1	386 (31)	57 (29)	0.002		
2	344 (27)	23 (12)	0.002		
3	189 (15)	23 (12)	0.002		
4	80 (6)	7 (4)	0.002		
5	28 (2)	3 (2)	0.002		
6	5 (<1)	2 (3)	0.002		

* Positive and negative likelihood ratios for predicting an abnormal TSH level (<0.3 or >5μIU/mL).

† Mean age for ≥0.3μIU was 68.9; median 70, IQR 60–79. Mean age for <0.3μIU was 67.4; median 68; IQR 59–78. p-value 0.13.

*TSH*, thyroid-stimulating hormone; +*LR*, positive likelihood ratio; −*LR*, negative likelihood ratio; *CHADS2*, Congestive heart failure, Hypertension, Age ≥75, Diabetes mellitus, Prior Stroke or Transient ischemic attack or Thrombeombolism

**Table 4:** Performance characteristics of a clinical decision rule for predicting low TSH (<3μIU) or any TSH abnormality (<0.3 or >5μIU/mL) in 1,984 patients presenting to the emergency department with atrial fibrillation.

Low TSH			Low or elevated TSH		
	
Decision rule	TSH <0.3μIU/mL	TSH ≥0.3μIU/mL	Decision rule	TSH abnormal	TSH normal
Yes	32	1031	Yes	145	918
No	4	391	No	50	345

* Sensitivity 88.9% (95% CI [73.0–96.4]), specificity 27.5% (95% CI [25.2–29.9]), PPV 3.0% (95% CI [2.1–4.3]), and NPV 99.0% (95% CI [97.2–99.7]). +LR 1.23 (95% CI [1.09–1.38]). −LR 0.40 (95% CI [0.16–1.02]).

† Sensitivity 74.4% (95% CI [67.5–80.2]), specificity 27.3% (95% CI [24.9–29.9]), PPV 13.6% (95% CI [11.7–15.9]), and NPV 87.3% (95% CI [83.6–90.4]). +LR 1.02 (95% CI [0.94–1.12]). −LR 0.94 (95% CI [0.74–1.20]).

*TSH*, thyroid-stimulating hormone; *+LR*, positive likelihood ratio; −*LR*, negative likelihood ratio; *PPV*, positive predictive value; *NPV*, negative predictive value
